# Development and Statistical Optimisation of Buspirone Hydrochloride Buccoadhesive Films

**DOI:** 10.1155/2014/214026

**Published:** 2014-10-30

**Authors:** Upendra Nagaich, Vandana Chaudhary, Jaya Nagaich

**Affiliations:** ^1^Department of Pharmaceutics, Amity Institute of Pharmacy, Amity University, Noida, Uttar Pradesh 201313, India; ^2^Department of Pharmaceutics, B. S. Anangpuria Institute of Pharmacy, Faridabad, India; ^3^Department of Pharmaceutics, Kota College of Pharmacy, Kota, India

## Abstract

The aim of the present study was to prepare unidirectional buccal films of buspirone hydrochloride by solvent casting technique. Hydroxypropylmethylcellulose (HPMC K15M) and Eudragit RL-100 were used as polymers in different proportion. Polyethylene glycol 400 and sodium lauryl sulphate were used as plasticizer and permeation enhancer, respectively, in different concentration. In the formulation, total amount of polymer (*X*_1_) and percentage of HPMC K15M (*X*_2_) were kept as independent variables. Afterwards, statistically optimized process was carried out and two optimized formulations (OF1 and OF2) were developed. The observed results of optimized formulation were showed a greater degree of percentage of similarity with predicted values. The stability studies showed that there was no significant change found in physicochemical properties, *in-vitro* release, and *ex-vivo* diffusion studies.

## 1. Introduction

The present paper deals with the planning and realization of mucoadhesive polymeric films containing an anxiolytic drug buspirone hydrochloride for the buccal administration in the oral cavity. The advantage resides on the reduction of dose of the drug and hepatic first pass metabolism because of its localization in the oral cavity for systemic release. One particular problem that is common to many drug delivery systems, aimed at the treatment of the oral cavity diseases, is the short residence time at the site of application. This problem may be resolved by using bioadhesive polymers, that is, polymers that exhibit characteristic adhesive interactions with biological membranes [1]. Various bioadhesive mucosal dosage forms including adhesive tablets, gels, and films have been developed [2]. However, buccal films are preferable over adhesive tablets in terms of flexibility and comfort. In addition, they can circumvent the relatively short residence time of oral gels on the mucosa, which are easily washed and removed by saliva. Moreover, buccal films are also suitable for protecting wound surfaces, thus reducing pain and increasing the treatment effectiveness [3]. In this study, mucoadhesive films were developed by using the solvent casting method. For this purpose, hydrophilic water-soluble film forming polymer HPMC K15M and hydrophobic water permeable polymer (Eudragit RL-100) for controlling rate of release of drug; thus, diffusion of drug was used. Polyethylene glycol (PEG) 400 was used as plasticizer and sodium lauryl sulphate was used as permeation enhancer in varying concentration. In order to prepare the films, film-forming polymers were initially used alone and successively in combination with water permeable polymer (Eudragit RL-100) for controlling the rate of drug release [4]. The films with the best results were selected on the basis of their* in-vitro*,* ex-vivo* and optimisation of various parameters like* T*_50%_ and* T*_80%_; diffusion at 3 h, 6 h, and 9 h were carried out. Buspirone hydrochloride was eventually introduced in the film; hence, formulations OF1 and OF2 closely met desired data and were able to diffuse continuously the drug for 12 h, which can maintain the desired therapeutic concentration in plasma.

## 2. Materials and Methods

### 2.1. Materials

Buspirone Hydrochloride was purchased from Sigma chemicals, Bangalore (India). HPMC K15M was received as gift sample from Central drug house (P) Ltd., Delhi, India, and Eudragit RL-100 also received as gift sample from Sun pharmaceuticals, Baroda, India. Polyethylene glycol (PEG 400) and sodium lauryl sulphate were received from Merck Ltd., Mumbai (India). The polymers were dissolved in solvent mixture of distilled water and alcohol.

### 2.2. Methods

#### 2.2.1. Preformulation Studies

*Solubility Studies*. Solubility may be defined as spontaneous interaction of two or more substances to form homogeneous dispersions. The solubility of buspirone hydrochloride was studied in various aqueous and nonaqueous solvents. 10 mg of drug was taken in 10 mL of each solvent at room temperature, in screw-capped test tubes and shaken for 24 h in wrist action shaker. The solubility was checked by UV spectroscopy at 254 nm [5].

*Partition Coefficient. *The partition coefficient directly influences the permeability through various membranes. The study has been designed to determine partition coefficient of drug in 1-octanol: phosphate buffer saline (PBS) pH 6.8 [6].

*Partition Coefficient between 1 Octanol: PBS (pH 6.8) Was Determined by the Following Method. *Equal volume (10 mL) of PBS (pH 6.8) and 1-octanol was added to 10 mg of accurately weighed drug buspirone hydrochloride in glass-stoppered tube. The mixture was shaken for 24 h on a wrist action shaker. The drug concentration in both aqueous and 1-octanol phases was determined spectrophotometrically at 250 nm after separating both phases and calculating the partition coefficient [6].

*Experimental Design Approach. *Experimental design is a statistical design that prescribes or advises a set of combination of variables. The number and layout of these design points within the experimental region depend on the number of the effects that must be estimated. Depending on the number of factors their levels, possible interactions, and order of the model, various experimental designs are chosen. Each experiment can be represented as a point within the experimental domain, the point being defined by its coordinate (the value given to the variables) in the space.

It is a 3^2^ full factorial experimental design, which uses dimensional factor space at the corner of the design space. A factorial design was used in experiment, the effects of different factors, or conditions on experimental results were elucidated. These were the design points for simultaneous determination of the effect of several factors and their interaction.

Where the three factors was considered, at two levels, nine experiments were necessary which are situated at the corners of an orthogonal cube in a three dimensional space. The numbers of experiments are given by 2^*n*^, where *n* is the number of factors. If the numbers of factors and levels are large, then the number of experiments needed to complete a factorial design is large.

The fitting of an empirical polynomial equation to the experimental result facilitates the optimisation procedures. The general polynomial equation is as follows:

(1) Y=B0+B1X1+B2X2+B3X3+⋯+B12X1X2+B13X1X3 +B23X2X3+⋯+B123X1X2X3,

where *Y* is the response (dependent variable) as shown in Table 1.

*X*_1_, *X*_2_, *X*_3_ were the independent variable (concentration) of the 1, 2, and 3 factors [7]: *B*_1_, *B*_2_, *B*_3_, *B*_12_, *B*_13_, *B*_23_, *B*_123_ is the polynomial coefficients. *B*_0_ is the intercept (which represents the response when the levels of all factors are low). Generation and evaluation of the statistical experimental design were performed with the Microsoft Excel 2002 Add-In, Essential Regression. The studied factors were of total weight of polymer, and percentage of HPMC K15M in the formulations, as these polymers and the level settings used were found to have a significant effect on mucoadhesion and drug release of the matrices in preliminary investigations. The response variables were optimize the maximum detachment force (MDF). A design matrix was comprising of nine experimental runs. Table 1 summarizes the factors and their levels.

Buccal films were prepared by solvent casting technique. HPMC K15M was used as a rate controlling polymer and Eudragit RL-100 was used as a film forming agent, in the preparation of films. Polyethylene glycol (PEG 400) was used as a plasticizer. Sodium lauryl sulphate was used as permeation enhancer. The polymers were dissolved in solvent mixture of water and alcohol. The drug was then dispersed uniformly in the viscous solution with continuous stirring. The resulting mass was poured onto the glass mould of 2 cm in diameter. The moulds were left undisturbed at room temperature for a day. The films could be retrieved intact by slowly lifting from the moulds. The composition of films was shown in Table 2, based on 3^2^ full factorial designs [8].

Ethyl cellulose 10% w/v was dissolved in ethanol and polyethylene glycol (PEG 400) as a plasticizer was added to formulate backing layer by casting 0.5 mL of the solution on the dry films [9].

#### 2.2.2. Evaluation Parameters

Physicochemical evaluations of buspirone hydrochloride films as shown in Table 3.

#### 2.2.3. Surface pH

The surface pH was determined by the method similar to that used by Ilango et al. [9]. A combined glass electrode was used for this purpose. The films were kept in contact with 0.5 mL of distilled water for 1 h. pH was noted by bringing the electrode near the surface of the formulations and allowing it to equilibrate for 1 min [9].

#### 2.2.4. Swelling Study

Three films of 2 cm^2^ of each formulation of different batches were accurately weighed (*W*_1_) and it was placed in petridish containing 25 mL distilled water. Films were carefully removed from the petridish after 10 min. and wiped by using tissue paper. The weight of the swollen film was noted (*W*_2_). The swelling index was calculated by the formula [9].

(2) $$\begin{array}{c} \text{Swelling}\;\text{index}=\frac{\left(W_{2}-W_{1}\right)}{W_{1}}, \end{array}$$

where *W*_1_ = dry weight of the film and *W*_2_ = wet weight of the film.

#### 2.2.5. Weight Uniformity

Three samples of films from each batch were randomly taken and weighed individually each film. The data were analyzed for mean weight and standard deviation [9].

#### 2.2.6. Film Thickness

Five samples from each batch were taken and thickness of each film was determined using “screw gauge.” The data were analyzed for mean film thickness and standard deviation, number of times repetition (*n*) = 5, detection limit: 0.05 mm [9].

#### 2.2.7. Folding Endurance

Folding endurance of the film was determined by repeatedly folding a small strip of film at the same place until it broke. The number of times the film could be folded at the same place without breaking gave the value of folding endurance [9].

#### 2.2.8. Evaluation of Buccal Films of Buspirone Hydrochloride

*Test for Drug Content Uniformity of Active Ingredient*. A 2 cm^2^ film was cut into small pieces, dissolved into 10 mL of methanol and diluted into 100 mL with the simulated salivary fluid at pH 6.8, and shaken continuously. The solution was then filtered and drug was estimated spectrophotometrically at 254 nm after dilution [10].

*In-Vitro Bioadhesive Studies. *The bioadhesive strength of the buccoadhesive patch with porcine mucosa was measured using modified two arm balance. One arm of the balance was replaced with a steel pan. Pig buccal mucosa was obtained from local slaughter house and stored in simulated saliva fluid of pH 6.8 at 4°C. The experiment was performed within three hours of procurement of the mucosa. The buccal mucosa was fixed at the bottom of a glass beaker of capacity 100 mL with cyanoacrylate adhesive and this assembly was put in a beaker of capacity 500 mL. Simulated saliva was added into the 500 mL beaker until the upper surface of buccal mucosa completely dipped in the simulated saliva fluid to maintain the viability of buccal mucosa during the experiment. The patch was attached to the steel pan of the apparatus, the beaker was raised slowly until contact between buccal mucosa, and patch was established. A preload of 20 gm was placed on clamp for 5 min (preload time) to establish adhesion bonding between patch and buccal mucosa. The preload and preload time were kept constant for all the formulations. After completion of preload time, preload was removed from clamp, and water was then added into the beaker from the burette [10].

The addition of water was stopped when patch just detached from buccal mucosa. The weight of water required to detach the patch from buccal mucosa was noted. This was taken as a measure of mucoadhesive strength. The experiment was repeated with fresh mucosa in an identical manner for all the patches. Bioadhesion force (kg m^−1^ s^2^) is given by following equation:

(3) $$\begin{array}{c} F=\frac{\left(W_{w}\times g\right)}{A}, \end{array}$$

where *F* is the bioadhesion force (kg m^−1^ s^2^), *W*_*w*_ is the weight of water added, *g* is the acceleration due to gravity (cm/s^2^), and *A* is the surface area of the patch (cm^2^).

*In-Vitro Release Studies by Using Dissolution Apparatus*. The drug release was determined using U.S.P. dissolution test apparatus, thermostated at 37 ± 2°C, and stirred at a rate of 50 rpm. Sink condition was maintained throughout the study. Each film was fixed on glass slide with the help of cyanoacrylate adhesive, so that the drug could be released only from upper face. The slide was immersed in the vessel containing 250 mL of simulated salivary fluid pH 6.8. Aliquots of 5 mL of sample were withdrawn with graduated pipette at every 1 h time intervals up to 9 h with equal volume of simulated salivary fluid [11].

The samples were diluted with simulated salivary fluid and analyzed spectrophotometrically at 254 nm and the cumulative amount of drug released at various time intervals was calculated. The test was carried out in triplicate.

*Ex-Vivo Diffusion Studies by Using Keshary-Chien (K-C) Diffusion Cell*.* Ex-vivo* diffusion study of pure drug was carried out using fresh porcine oral mucosa tissue, placed in Krebs buffer pH 7.4. Isolation of the epithelium was done mechanically by using scissors and forceps. These studies were carried out using modified K-C diffusion cell. A buccal patch was placed over it to secure the patch tightly and prevent it from getting dislodge from the membrane [12].

The donor compartment was filled with 10 mL of simulated salivary fluid pH 6.8 containing 20% of methanol. The receptor compartment contained 25 mL of phosphate buffer pH 7.4 having 20% methanol to maintain sink conditions. The hydrodynamics in receptor compartment was maintained by stirring with a magnetic bead at 50 rpm. The whole assembly was maintained at 37 ± 2°C. 1 mL of samples was withdrawn from receptor compartment at every 1 h time interval up to 12 h and replaced with the same amount of fresh medium. The withdrawn samples were then diluted suitably, and analyzed spectrophotometrically at 254 nm, and percentage cumulative drug diffused (Table 4).

*Analysis of Release Mechanism*. The* in-vitro* release data were treated by different equations and kinetic models to explain the release kinetics of buspirone hydrochloride from the buccal patches [12].

The kinetic models used were zero order equations and first order equations (Higuchi release, Korsemeyer and Peppas models [13]).

*Statistical Optimisation. *Statistical experimental designs have been in use for several decades. These experimental layouts can be adopted at various phases of an optimisation process, such as in screening experiments or in finding the optimal conditions for targeted results. The results of statistically planned experiment are better accepted than those of traditional single-variable experiments. Response surface, contour surfaces methodology is now established as a convenient method for developing optimum processes with precise conditions and has minimized the cost of production of many a process with efficient screening of process parameter [14–16].

#### 2.2.9. Stability Studies

The purpose of stability testing is to provide evidence on how the quality of a drug substance or drug product varies with time under the influence of a variety of environmental factors such as temperature, humidity, and light, and to establish a retest period for the drug substance or a shelf life for the drug product and recommended storage conditions. To assess the drug and formulation stability, stability studies were done according to ICH guidelines Q1C.

Short-term stability studies were carried out on the films of most satisfactory as per ICH Guidelines Q1C. The most satisfactory formulation stored in sealed in aluminum foil. These were stored at 30 ± 2°C (65 ± 5% RH) and 40 ± 2°C (75 ± 5% RH) for 2 months. Films were evaluated for physical characteristics, buccoadhesive properties,* in-vitro* drug release study and* ex-vivo* diffusion study.

## 3. Results and Discussion

The sample of buspirone hydrochloride was identified and characterized as per the identification test in official monograph. The physical appearance of drug under investigation found similarity with official monograph. The drug sample complied with results of identification tests as reported in official monograph. The drug purity was identified by Infrared spectroscopy and characteristic peaks were obtained in the spectra for the different functional groups. The solubility profile of drug was determined in aqueous and nonaqueous solvents, buspirone hydrochloride is slightly soluble in ethanol, methanol, freely soluble in phosphate buffer pH 7.4, and water and soluble in 0.1 N HCl. The partition coefficient was found to be 2.7 in 1 octanol: PBS (pH 6.8). This indicated that the drug has an adequate hydrophilic and lipophilic balance.

### 3.1. Formulation Studies

Buccal films of buspirone hydrochloride were prepared by solvent casting technique. In this design total amount of polymer and percentage of HPMC K15M were taken as independent variables. Amount of sodium lauryl sulphate and polyethylene glycol 400 were kept constant. Formulations were shown in the Table 2.

### 3.2. Evaluation Parameters

#### 3.2.1. Surface pH

An acidic or alkaline formulation is bound to cause irritation on the mucosal membrane and hence this parameter assumes significance while developing a buccoadhesive formulation. Surface pH of the formulation F1 to F9 was varied from 6.3 ± 0.02 to 6.7 ± 0.05. Each sample was analyzed in triplicate (*n* = 3). The results were revealed that all the formulations provide an acceptable pH in the range of 6.2 to 6.8 (salivary pH). Hence, they may not produce any local irritation to the mucosa as shown in Table 3.

#### 3.2.2. Swelling Studies

Any polymer with good swelling property is expected to be a good candidate for bioadhesive application. When bioadhesive is exposed to aqueous medium, they swell and form a gel. The rate and extent of water uptake by a polymer has been reported to be an important factor in determination of its relative bioadhesive strength. Uptake of water results in relaxation of originally stretched, entangled, (or) twisted polymer chain, resulting in exposure of all polymer bioadhesive sites for bonding to occur. The faster this phenomenon occurs the more rapid will be the polymer films adherance to its substrate. The results were revealed that all the formulations provide an acceptable swelling index in the range of 1.09–1.59 as shown in Table 3.

#### 3.2.3. Weight Uniformity

Weight Uniformity of HPMC K15M and Eudragit RL 100 based formulations F1–F9 varied from 146.1 to 456.1 mg as shown in Table 3.

#### 3.2.4. Thickness

As the total amount of polymer increases the thickness of films were found to be increased. The formulation F7 showed the lowest thickness (91.74 ± 0.7 *μ*m) while formulation F3 showed highest thickness (314.22 ± 2.6 *μ*m) as shown in Table 3.

#### 3.2.5. Folding Endurance

As the amount of HPMC K15M (film forming polymer) increases, the folding endurance was found to be increase. Therefore, formulation F3 showed greater folding endurance (68 ± 4) compared to other formulations as shown in Table 3.

#### 3.2.6. Drug Content

Drug content for all the formulations was found to be between 98 and 99%, which within the desirable range as shown in Table 3.

#### 3.2.7. *In-Vitro* Bioadhesion

As the amount of HPMC K15M increases, the* in-vitro *bioadhesion was found to be increase. Therefore, formulation F3 showed a greater bioadhesion strength (13.67 ± 0.49 g) as shown in Table 3.

#### 3.2.8. *In-Vitro* Drug Release Studies

**Figure 1:**
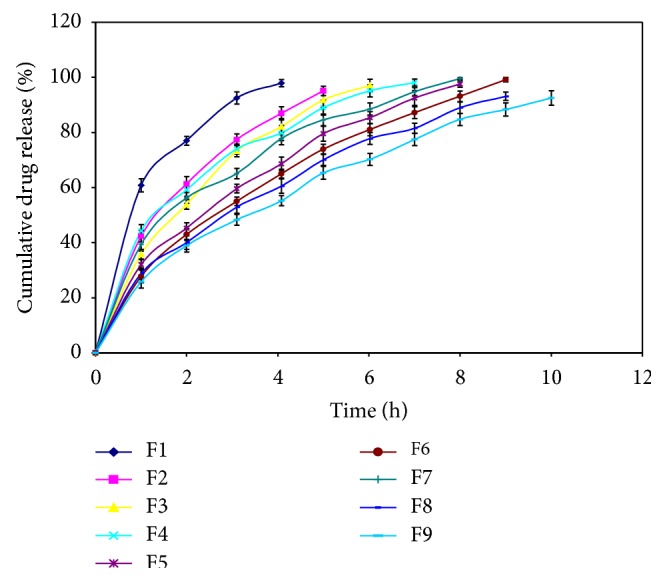
*In-vitro* drug release studies of various formulations.

In the formulations, F1 to F3, having HPMC K15M alone, gave faster drug release as compared to other formulations, which had HPMC K15M in combination with Eudragit RL-100, which would retarded drug release from the buccal films. Formulation F1 releases 99% drug within 4 h, while formulation F9 releases 88% drug within 9 h as shown in Figure 1.

#### 3.2.9. *Ex-Vivo* Drug Diffusion Studies

**Figure 2:**
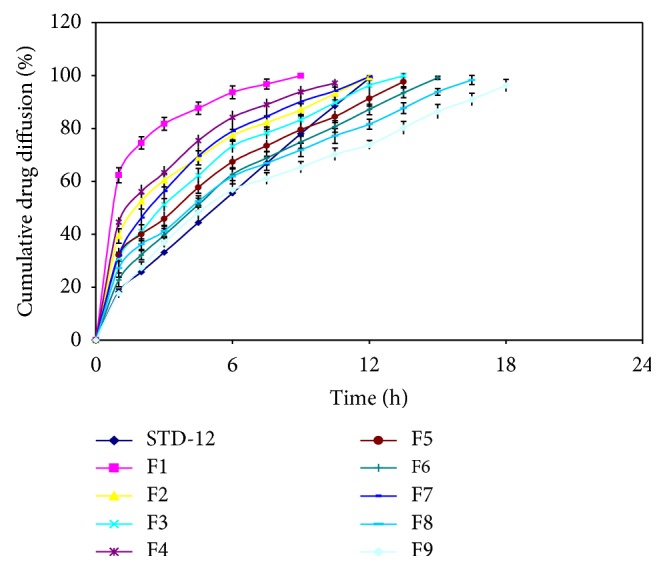
*Ex-vivo* drug diffusion studies of various formulations.

In the formulations, F1 to F3, having HPMC K15M alone, gives faster drug diffusion as compared to other formulations, which had HPMC K15M in combination with Eudragit RL-100, which retarded drug diffusion from the buccal films. Formulation F1 diffuses 99% drug within 9 h, while formulation F7 diffuses 99% drug within 12 h and formulation F9 diffuses 74% within 12 h as shown in Figure 2.

#### 3.2.10. Analysis of Release Mechanism

Values of *R* ^2^ were all close to unity, indicating that the first order release behavior is the main release mechanism. Hence release pattern from buccal films are diffusion, in which firstly films were swelled then released slowly as shown in Table 4.

#### 3.2.11. Fitting of the Data to the Model

A positive value represents an effect that favors the optimization, while the negative value indicates an inverse relationship between factors and responses. It was concluded from the above equation that two variables, that is, total amount of polymer and amount of HPMC K15M, have a greater positive value in equation obtained for 9 h* ex-vivo* studies.

Coefficient factor in the regression equation represents interaction between variables. The independent variables change regularly so that factors can reproduce favorable results. Because there are higher positive values of *X*_1_,  *X*_2_,  *X*_1_ *X*_2_,  *X*_2_^2^ obtained in the equation at 9 h for response surface, this was taken as optimum.

From the fitting of the data to the model, it is clear that data should be selected from 9 h* ex-vivo* studies.

#### 3.2.12. Contour Plot and Response Surface Analysis

The two-dimensional and three-dimensional response plots were plotted which were very useful in the study in the interaction effect of the factors on the response. These types of plots useful in the study of the effects of factors on the response at one time. In the entire graph, three variables were kept constant and two variables were changed continuously at different levels so that desired result can be obtained.

The 9 h* ex-vivo* studies graph showed optimum responses for the formulation. It was showed linear relationship with statistical calculations as shown in Figures 3, 4, 5, 6, 7, 8, 9, 10, 11, and 12.

#### 3.2.13. Optimisation

*T*_50%_. One has

(4) Y=0.2641145∗X1−0.0135311∗X2−0.0005793∗X1X2 −0.002202∗X12−0.0003349∗X22.

*T*_80%_. One has

(5) Y=0.6412898∗X1−0.0447662∗X2−0.0003357∗X1X2 −0.0070657∗X12−0.000959∗X22.

*3 h*. One has

(6) Y=97.50941−1.41006∗X1+0.98072∗X2−0.0112013 ∗X1X2+0.0252299∗X12+0.0114420∗X22.

*6 h*. One has

(7) Y=97.967623+2.4813106∗X1+0.0150901∗X2 +0.0019077∗X1X2+0.026712∗X12 +0.0025469∗X22.

*9 h*. One has

(8) Y=97.996144−2.424963∗X1+0.2108694∗X2 +0.0059851∗X1X2+0.0212542∗X12 +0.0003545∗X22.

#### 3.2.14. Optimisation

The optimum formula was selected, based on* ex-vivo* studies. Upon trading of various response variables and comprehensive evaluation of feasibility search, the formulation composition with total amount of polymer was 400 mg, 350 mg, and for HPMC K15M is 75% w/w, 70% w/w for both optimized batches respectively. These were found to fulfill the maximum requisites for optimum formulation.

Hence, optimized formula was prepared by optimum amount of HPMC K15M and Eudragit RL-100, as per value obtained above as shown in Table 5.

### 3.3. Evaluation of Optimized Formulation

#### 3.3.1. Surface pH

Surface pH of the optimized formulation OF1 and OF2 displayed a pH of 6.2 ± 0.03 and 6.4 ± 0.04, respectively, which was within desirable range as shown in Table 6.

#### 3.3.2. Swelling Studies

The results revealed that optimized formulations provided an acceptable swelling index in the range of 1.17–1.21 as shown in Table 6.

#### 3.3.3. Weight Uniformity

Weight uniformity of HPMC K15M and Eudragit RL-100 based optimized formulations OF1 and OF2 showed (413.21 ± 2.66) mg and (353.47 ± 3.48) mg, respectively, as shown in Table 6.

#### 3.3.4. Thickness

Thickness of optimized formulations OF1 and OF2 was 292.52 ± 1.65 *μ*m and 259.69 ± 2.23 *μ*m, respectively, as shown in Table 6.

#### 3.3.5. Folding Endurance

Folding endurance of optimized formulation OF1 and OF2 was 144 ± 3 and 138 ± 4, which are within desirable range as shown in Table 6.

#### 3.3.6. Drug Content

Drug content of optimized formulations was found to be between 98 and 99%, which was within the desirable range as shown in Table 6.

#### 3.3.7. *In-Vitro* Bioadhesion

*In-vitro *Bioadhesion of optimized formulation OF1 and OF2 was found to be between 9 and 10 g, which is within the desirable range as shown in Table 6.

#### 3.3.8. *In-Vitro* Drug Release Studies

**Figure 13:**
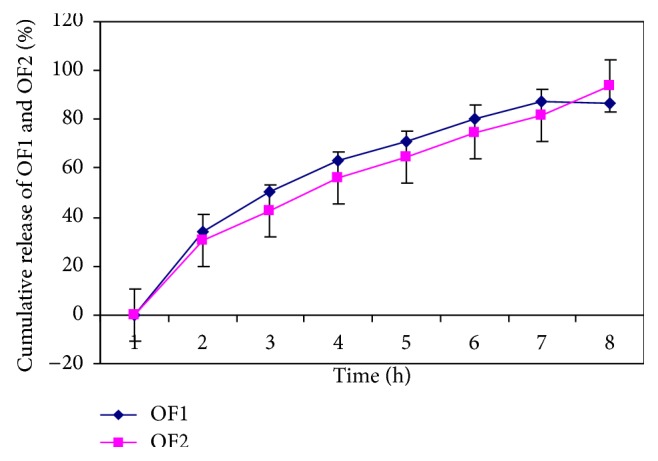
*In-vitro* drug release profile of optimized formulation OF1 and OF2.

Optimized formulations OF1 and OF2 release 98% drug within 6 h and 9 h, respectively, as shown in Figure 13.

#### 3.3.9. *Ex-Vivo* Drug Diffusion Studies

**Figure 14:**
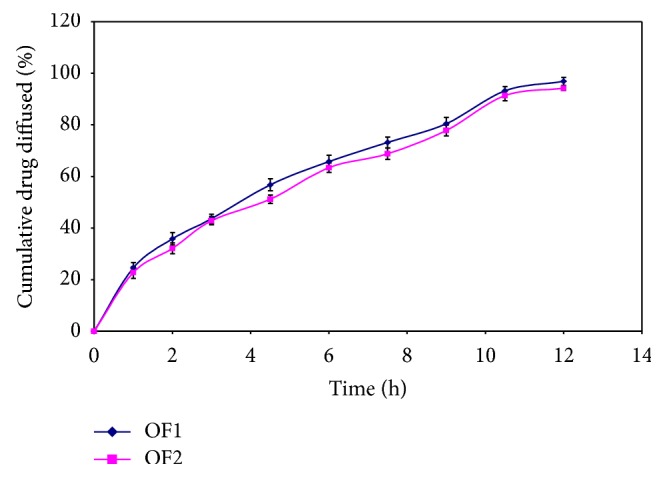
*Ex-vivo* drug diffusion profile of optimized formulations OF1 and OF2.

Optimized formulations OF1 and OF2 diffuse drug 96.87 ± 1.55% and 94.16 ± 2.13%, respectively, as shown in Figure 14.

#### 3.3.10. Statistical Analysis-Comparison of Various Variables

It showed that experimental values of* T*_50%_ and* T*_80%_, diffusion at 3 h, diffusion at 6 h, and diffusion at 9 h. OF1 and OF2 were near to expected values of OF1 and OF2, respectively, and also significant by similar desirable data as shown in Table 7.

#### 3.3.11. Stability Studies

**Figure 15:**
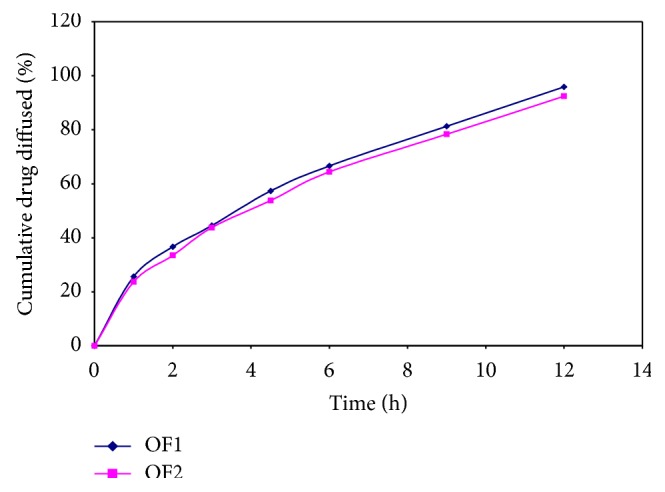
Plot of* ex-vivo* diffusion profile of optimized formulations at 30°C ± 2°C (65% ± 5% RH) after 60 days.

**Figure 16:**
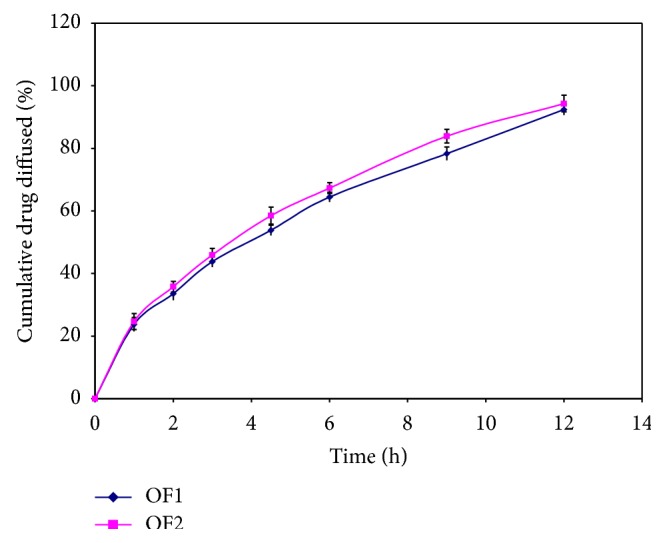
Plot of* ex-vivo* diffusion profile of optimized formulations at 40°C ± 2°C (75% ± 5% RH) after 60 days.

From the stability studies, it was concluded that no significant changes were found in the physicochemical parameters,* ex-vivo* drug diffusion profile of both optimized formulation after stability studies is shown in Tables 8 and 9 and in Figures 15 and 16.

#### 3.3.12. Scanning Electron Microscopy

**Figure 17:**
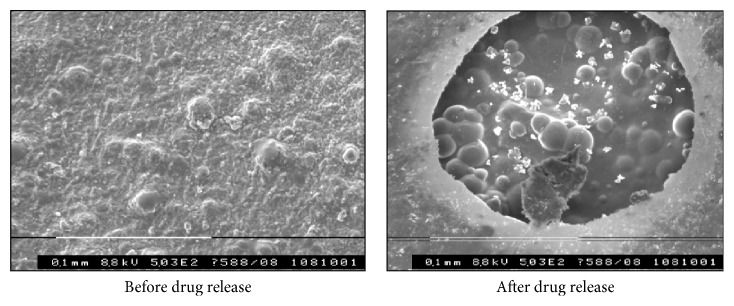
Scanning electron micrographs of surfaces of drug-containing buccal films of OF2 formulation.

Surface topography of buccal films was carried out. SEM study showed that erosion was found on the surface of buccal films, which indicates that drug was released by diffusion mechanism as shown in Figure 17.

## 4. Conclusion

In the present study, an attempt was made to design buccal films of buspirone Hydrochloride for treatment/management of anxiety. The main interest in such a dosage form was to formulate buccal films of buspirone Hydrochloride in order to increase bioavailability by avoiding first-pass metabolism and to control drug release in therapeutic range for longer period. Buccal films of buspirone hydrochloride were prepared by solvent casting technique using 3^2^ full factorial designs. In this dosage form, hydrophilic water-soluble film forming polymer HPMC K15M and hydrophobic water permeable polymer (Eudragit RL-100) for controlling the diffusion of drug. Optimisation of various parameters like* T*_50%_ and* T*_80%_ and diffusion at 3 h, 6 h, and 9 h were carried out and optimized formulations were developed. Results of optimized formulations OF1 and OF2 closely met targeted data and continuously diffused drug for 12 h, which can maintain desired therapeutic concentration in plasma. Stability study of optimized formulations OF1 and OF2 was performed which showed slight change in physicochemical parameters and drug diffusion profile.

Hence, from the above all discussion it is concluded that OF1 and OF2 formulation proved to be the best formulations, which have increased bioavailability and prolonged release time.

## Figures and Tables

**Figure 3:**
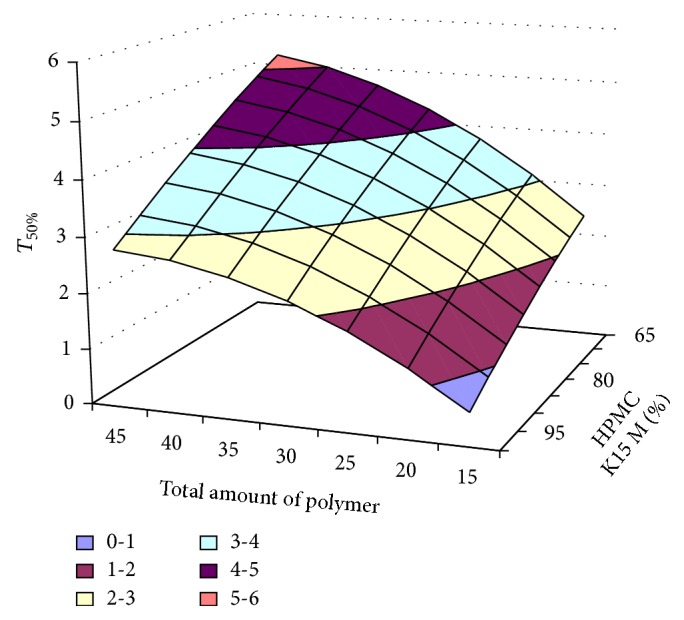
Surface plot of* T*_50%_ for the optimization of amount of HPMC K15M and total amount of polymer.

**Figure 4:**
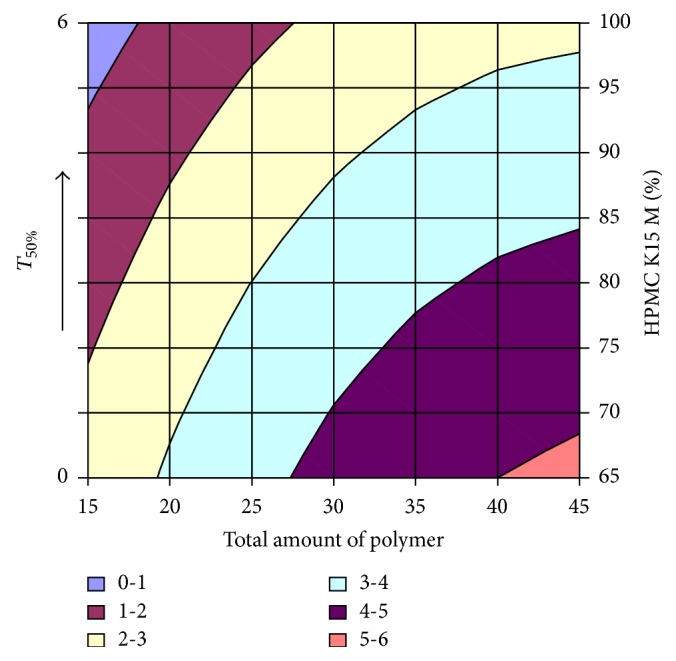
Contour plot of* T*_50%_ for the optimization of amount of HPMC K15M and total amount of polymer.

**Figure 5:**
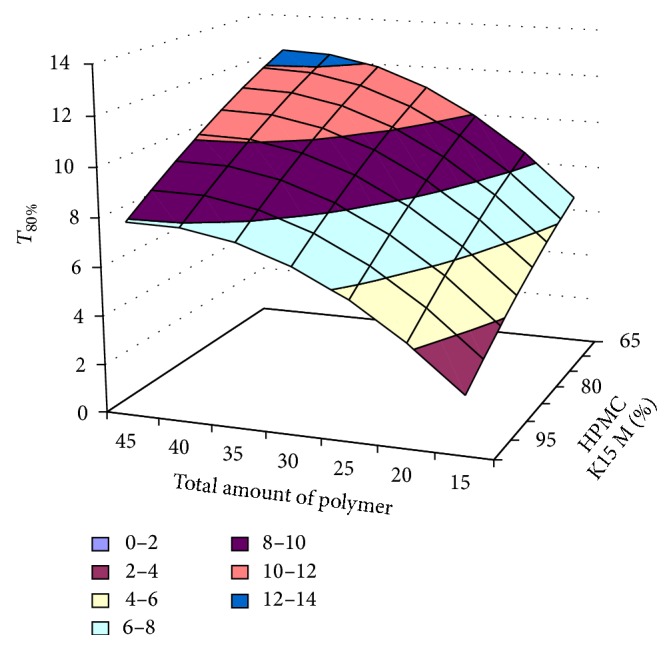
Surface plot of* T*_80% _for the optimization of amount of HPMC K15M and total amount of polymer.

**Figure 6:**
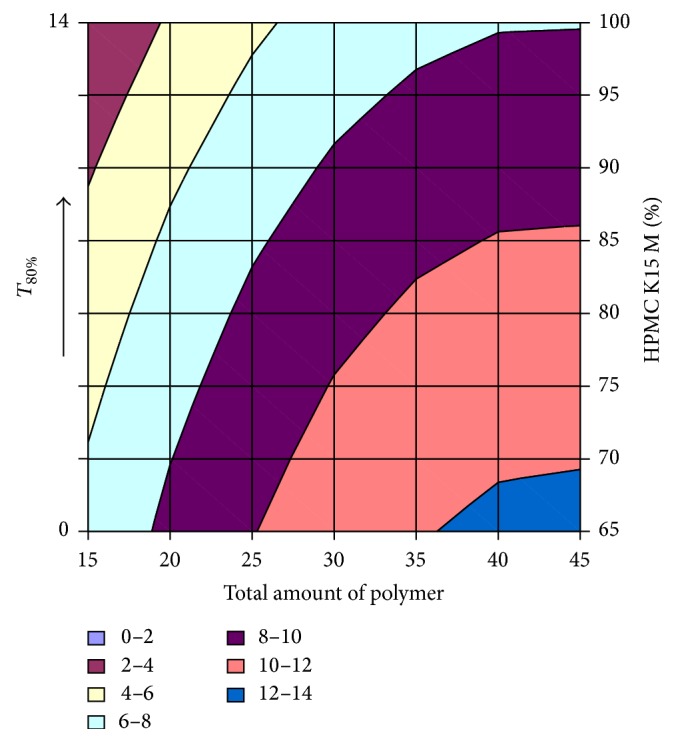
Contour plot of* T*_80%_ for the optimization of amount of HPMC K15M and total amount of polymer.

**Figure 7:**
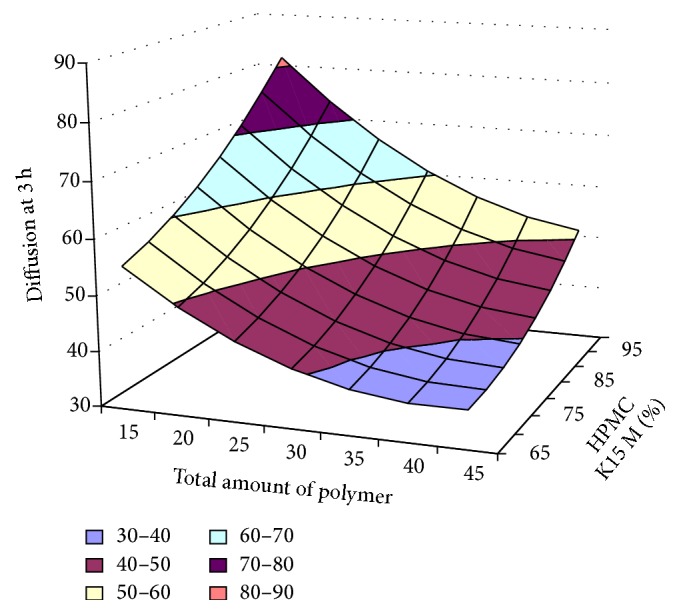
Surface plot diffusion at 3 h for the optimization total amount of polymer and HPMC K15M.

**Figure 8:**
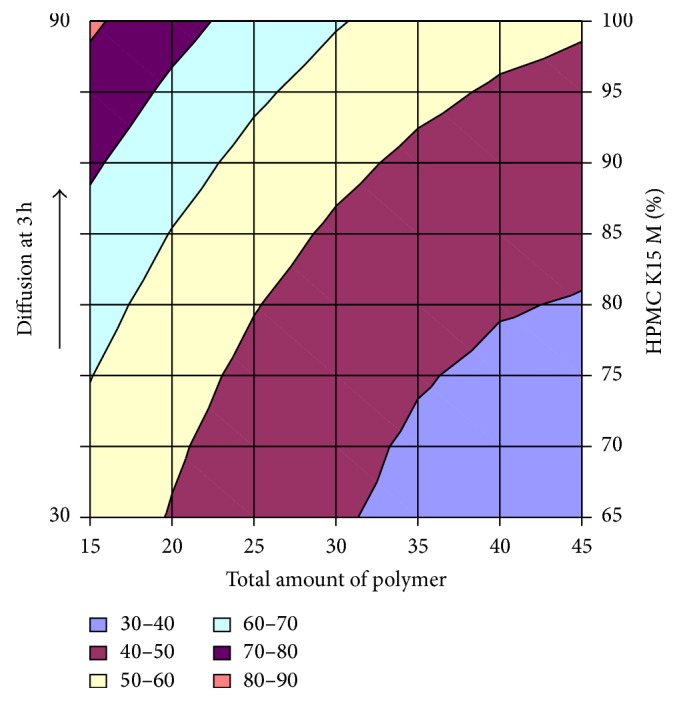
Contour plot of diffusion at 3 h for the optimization total amount of polymer and HPMC K15M.

**Figure 9:**
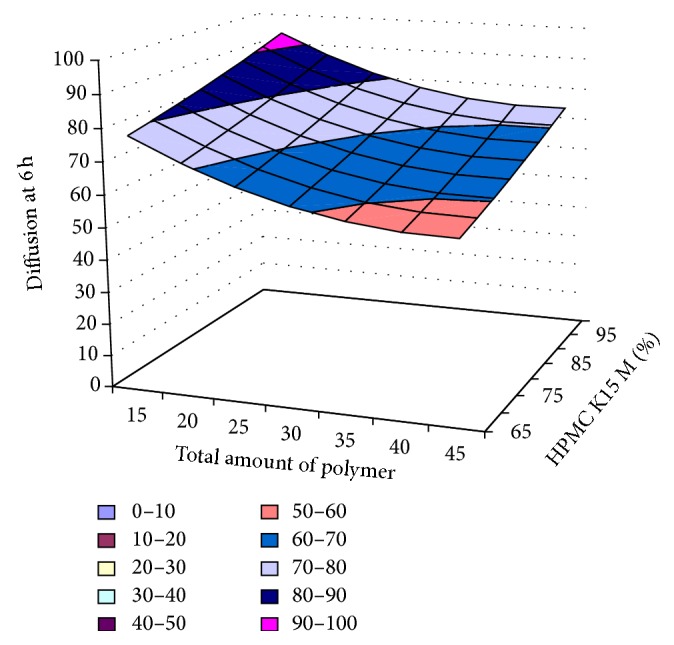
Surface plot of diffusion at 6 h for the optimization total amount of polymer and HPMC K15M.

**Figure 10:**
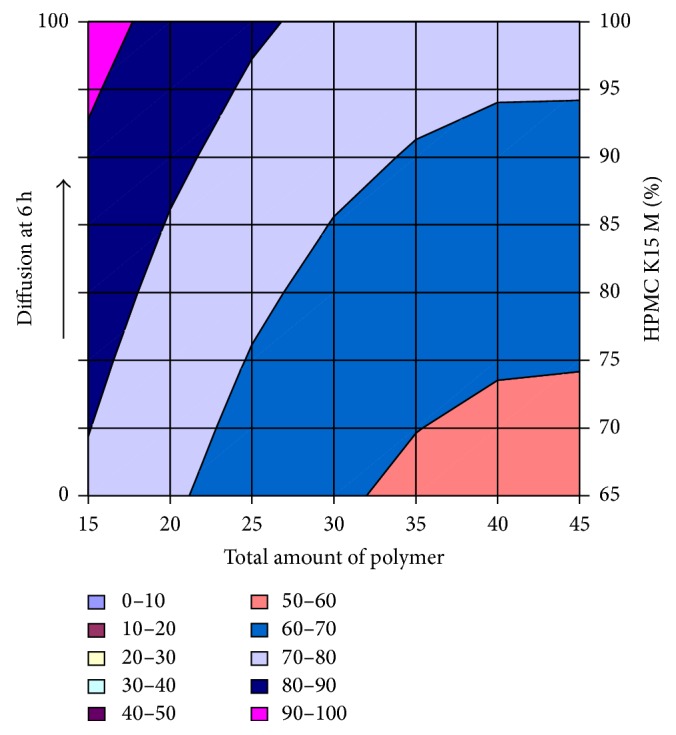
Contour plot of diffusion at 6 h for the optimization total amount of polymer and HPMC K15M.

**Figure 11:**
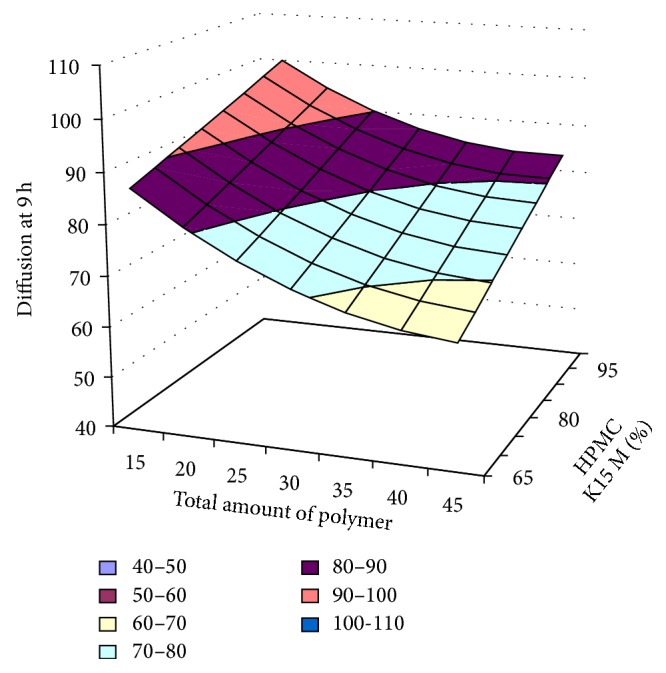
Surface plot of diffusion at 9 h for the optimization total amount of polymer and HPMC K15M.

**Figure 12:**
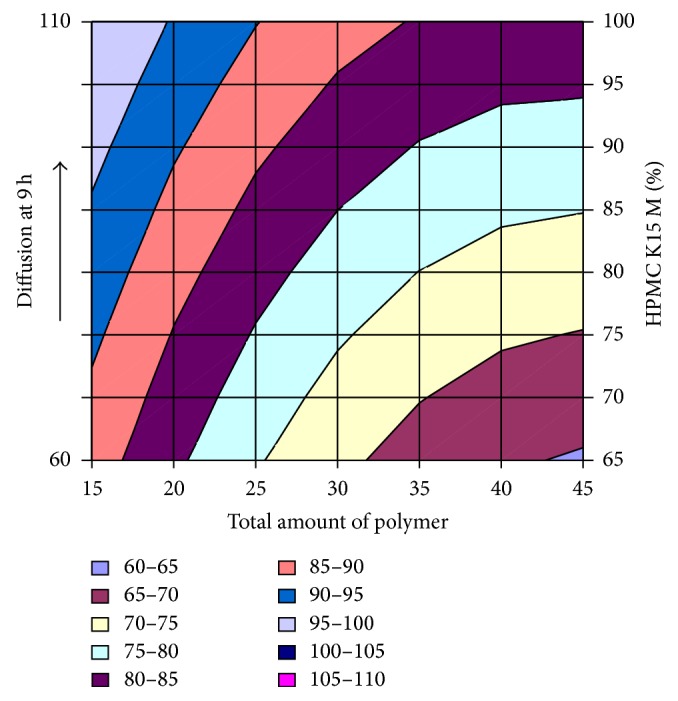
Contour plot of diffusion at 9 h for the optimization total amount of polymer and HPMC K15M.

**Table 1:** Independent variables and their levels.

Variables (code)	Level		
	−1	0	1
Total amount of polymer (mg) *X*_1_	15	30	45
Percentage of HPMC K15M (%) *X*_2_	66.66	80	100

**Table 2:** 3^2^ full factorial design for the formulation of bucco-adhesive films of Buspirone Hydrochloride.

Formulation code	Amount of drug	Total amount of polymer	Amount of HPMC K15M		Amount of Eudragit RL-100		Amount of permeation enhancer		Amount of plasticizer		Solvents	
		*X*_1_	*X*_2_				Na-lauryl sulphate		PEG 400		Water	Alcohol
	In mg		In %	In mg	In %	In mg	In %	In mL	In %	In mL	In mL	In mL
F1	50	150	100	150	0	0	5	0.009	20	0.03	15	0
F2	50	300	100	300	0	0	5	0.018	20	0.06	30	0
F3	50	450	100	450	0	0	5	0.027	20	0.09	45	0
F4	50	150	80	120	20	30	5	0.009	20	0.03	12	7.5
F5	50	300	80	240	20	60	5	0.018	20	0.06	24	15
F6	50	450	80	360	20	90	5	0.027	20	0.09	36	27.5
F7	50	150	66.66	100	33.33	50	5	0.009	20	0.03	10	12.5
F8	50	300	66.66	200	33.33	100	5	0.018	20	0.06	20	25
F9	50	450	66.66	300	33.33	150	5	0.027	20	0.09	30	37.5

**Table 3:** Physicochemical parameters of various formulations of buccal films.

Formulation code	Surface pH^*^	Swelling studies	Weight uniformity (mg)^*^	Thickness (*µ*m)^*^	Folding endurance^*^	Drug content^*^	Bioadhesion strength^*^ (g)
F1	6.3 ± 0.02	1.22	155.2 ± 3.56	124.74 ± 1.5	29 ± 4	98.12 ± 1.26	8.87 ± 0.12
F2	6.6 ± 0.04	1.37	303.1 ± 4.78	211.48 ± 2.2	46 ± 3	98.17 ± 0.96	10.19 ± 0.34
F3	6.7 ± 0.03	1.59	456.1 ± 6.13	314.22 ± 2.6	68 ± 4	99.07 ± 0.65	13.67 ± 0.49
F4	6.3 ± 0.04	1.09	153.2 ± 1.92	104.94 ± 0.8	21 ± 4	98.34 ± 1.37	7.38 ± 0.53
F5	6.6 ± 0.02	1.25	292.7 ± 3.87	202.88 ± 1.6	34 ± 4	98.67 ± 1.16	9.61 ± 0.48
F6	6.3 ± 0.03	1.41	441.2 ± 4.27	309.82 ± 1.8	53 ± 5	98.47 ± 1.27	11.39 ± 0.65
F7	6.6 ± 0.04	1.01	146.1 ± 1.69	91.74 ± 0.7	19 ± 3	98.05 ± 1.22	6.93 ± 0.81
F8	6.7 ± 0.05	1.12	305.3 ± 2.45	193.48 ± 1.3	31 ± 5	98.21 ± 1.34	8.08 ± 0.69
F9	6.5 ± 0.02	1.27	447.1 ± 4.43	295.22 ± 1.9	41 ± 5	98.19 ± 1.26	9.23 ± 0.78

^*^Data represents mean ± SD, *n* = 3.

**Table 4:** Diffusion kinetic profile of various formulations.

Kinetic profile of formulations	For Higuchi equation		For Peppas Korsmayer equation		For 1st order equation			For zero order equation	
	N	R^2^	N	log⁡*K*	N	R^2^	K	K	*R* ^2^
Desirability	25.130	0.912	0.698	1.218	−0.108	0.750	−0.249	8.926	0.961

F1	44.862	0.801	0.230	1.794	−0.214	0.907	−0.492	15.27	0.039
F2	30.364	0.900	0.369	1.595	−0.129	0.883	−0.298	9.98	0.467
F3	28.391	0.988	0.462	1.487	−0.102	0.955	−0.235	9.450	0.676
F4	32.988	0.799	0.319	1.661	−0.134	0.979	−0.308	10.41	0.299
F5	26.614	0.975	0.417	1.494	−0.082	0.968	−0.188	8.856	0.659
F6	24.497	0.994	0.549	1.347	−0.069	0.984	−0.160	8.255	0.818
F7	30.028	0.970	0.456	1.518	−0.131	0.903	−0.301	9.958	0.626
F8	24.480	0.992	0.459	1.422	−0.067	0.975	−0.154	8.185	0.728
F9	22.350	0.985	0.603	1.261	−0.057	0.983	−0.132	7.558	0.849

*N*: release exponent, *R*: correlation coefficient, log⁡*K*: slope.

**Table 5:** Formulation chart for optimized formulation.

Formulation code	Amount of drug (mg)	Total amount of polymer *X*_1_	Amount of HPMC K15M *X*_2_		Amount of Eudragit RL-100		Amount of permeation enhancer		Amount of plasticizer		Solvent	
							Na-lauryl sulphate		PEG 400		Water	Alcohol
			(%)	(mg)	(%)	(mg)	(%)	(mL)	(%)	(mL)	(mL)	(mL)
OF1	50	400	75	300	25	100	5	0.024	20	0.08	30	20
OF2	50	350	70	245	30	105	5	0.021	20	0.07	24.5	17.5

**Table 6:** Physicochemical parameters of optimized formulations.

Formulations	Surface pH^*^	Swelling studies	Weight uniformity (mg)^*^	Thickness (*µ*m)^*^	Folding endurance^*^	Drug content^*^	Bioadhesion strength^*^ (g)
OF1	6.2 ± 0.03	1.21	413.21 ± 2.66	292.52 ± 1.65	144 ± 3	98.42 ± 1.36	9.93 ± 0.34
OF2	6.4 ± 0.04	1.17	353.47 ± 3.48	259.69 ± 2.23	138 ± 4	98.07 ± 0.86	9.21 ± 0.58

^*^Data represents mean ± SD, *n* = 3.

**Table 7:** Comparison of various dependent variables of optimized formulations.

Parameters	Total Amount of Polymer	Percentage Of HPMC K15M	*T*_50%_ (h)	*T*_80%_ (h)	Diffusion at 3 h	Diffusion at 6 h	Diffusion at 9 h
Codes	*X*_1_	*X*_2_	*Y*_1_	*Y*_2_	*Y*_3_	*Y*_4_	*Y*_5_

Desirability			5.25	9.28	33.18	55.60	78.01

Predicted OF1	400	75	4.43	11.30	38.68	60.63	70.63
Predicted OF2	350	70	4.43	11.40	39.04	60.14	70.20
Experimental OF1	400	75	4.12	8.56	45.56 ± 1.78	65.78 ± 2.42	80.42 ± 2.44
Experimental OF2	350	70	4.41	9.23	42.87 ± 1.54	63.45 ± 1.84	77.84 ± 2.20

**Table 8:** Physicochemical properties of optimized formulations during the stability studies.

Formulation code		OF1			OF2		
Time (Days)		0	30	60	0	30	60
Surface pH	A	6.1 ± 0.02	6.1 ± 0.03	6.2 ± 0.04	6.3 ± 0.04	6.4 ± 0.05	6.4 ± 0.07
	B	6.1 ± 0.04	6.2 ± 0.03	6.3 ± 0.05	6.3 ± 0.04	6.4 ± 0.03	6.4 ± 0.07

Swelling studies	A	1.18	1.18	1.17	1.13	1.12	1.12
	B	1.18	1.17	1.17	1.13	1.11	1.08

Weight uniformity	A	435.70 ± 1.59	426.79 ± 2.28	423.54 ± 2.65	384.39 ± 2.38	378.47 ± 3.32	375.76 ± 2.29
	B	435.70 ± 1.59	423.86 ± 2.18	421.65 ± 2.58	384.39 ± 2.38	372.71 ± 1.63	370.34 ± 2.34

Thickness (*µ*m)^*^	A	283.35 ± 1.54	282.45 ± 1.65	282.13 ± 1.87	251.56 ± 1.97	249.31 ± 2.12	249.06 ± 2.33
	B	283.35 ± 1.54	280.76 ± 1.32	280.45 ± 1.21	251.56 ± 1.97	247.12 ± 3.34	246.41 ± 3.12

Folding endurance^*^	A	134 ± 2	131 ± 3	129 ± 4	126 ± 3	122 ± 4	121 ± 5
	B	134 ± 2	130 ± 4	128 ± 4	126 ± 3	121 ± 3	119 ± 5

Drug content^*^	A	96.21 ± 2.47	95.17 ± 3.12	93.28 ± 2.27	95.53 ± 1.96	93.29 ± 2.78	91.18 ± 2.65
	B	96.21 ± 2.47	93.34 ± 2.19	92.19 ± 2.43	95.53 ± 1.96	92.67 ± 2.79	90.29 ± 2.52

A: 30°C ± 2°C/65% ± 5% RH; B: 40°C ± 2°C/75% ± 5% RH.

^*^Data represents mean ± SD, *n* = 3.

**Table 9:** *In-vitro* bioadhesive strength of optimized formulations.

Formulations	Bioadhesion strength^*^ (g)	
OF1	A	8.85 ± 1.56
	B	8.18 ± 1.27

OF2	A	8.27 ± 1.29
	B	7.85 ± 1.17

A: 30°C ± 2°C (65% ± 5% RH); B: 40°C ± 2°C (75% ± 5% RH).

^*^Data represents mean ± SD, *n* = 3.
